# Sequence to structure analysis of the ORF4 protein from Hepatitis E virus

**DOI:** 10.6026/97320630017818

**Published:** 2021-09-30

**Authors:** Zoya Shafat, Anwar Ahmed, Mohammad K Parvez, Shama Parveen

**Affiliations:** 1 Centre for Interdisciplinary Research in Basic Sciences, Jamia Millia Islamia, New Delhi, India; 2 Centre of Excellence in Biotechnology Research, College of Science, King Saud University, Riyadh, Saudi Arabia; 3 Department of Pharmacognosy, College of Pharmacy, King Saud University, Riyadh, Saudi Arabia

**Keywords:** Hepatitis E virus (HEV), open-reading frame 4 (ORF4), mutational analysis, entropy analysis, gene selection pressure, nucleotide diversity, transition/transversion ratio, structural analysis, intrinsic disordered proteins (IDPs), drug target

## Abstract

Hepatitis E virus (HEV) is the main cause of acute hepatitis worldwide. HEV accounts for up to 30% mortality rate in pregnant women, with highest incidences reported for genotype 1 (G1) HEV. The contributing factors in adverse cases during pregnancy in women due to HEV infection is still debated. The mechanism underlying the pathogenesis of viral infection is attributed to different genomic component of HEV, i.e., open reading frames (ORFs): ORF1, ORF2, ORF3 and ORF4. Recently, ORF4 has been discovered in enhancing the replication of GI isolates of HEV through regulation of an IRES-like RNA element. However, its characterization through computational methodologies remains unexplored. In this novel study, we provide comprehensive overview of ORF4 protein's genetic and molecular characteristics through analyzing its sequence and different structural levels. A total of three different datasets (Human, Rat and Ferret) of ORF4 genomes were built and comparatively analyzed. Several non-synonymous mutations in conjunction with higher entropy values were observed in rat and ferret datasets, however, limited variation was observed in human ORF4 genomes. Higher transition to tranversion ratio was observed in the ORF4 genomes. Studies have reported the association of intrinsic disordered proteins (IDP) with drug discovery due to its role in several signaling and regulatory processes through protein-protein interactions (PPIs). As PPIs are potent drug target sources, thus the ORF4 protein was explored by analyzing its polypeptide structure in order to shed light on its intrinsic disorder. Pressures that lead towards preponderance of disordered-promoting amino acid residues shaped the evolution of ORF4. The intrinsic disorder propensity analysis revealed ORF4 protein (Human) as a highly disordered protein (IDP). Predominance of coils and lack of secondary structure further substantiated our findings suggesting its involvement in binding to ligand molecules. Thus, ORF4 contributes to cellular signaling processes through protein-protein interactions, as IDPs are targets for regulation to accelerate the process of drug designing strategies against HEV infections.

## Background:

Hepatitis E virus (HEV) is a major causative agent of viral hepatitis transmitted enterically worldwide. HEV is the major aetiological agent of Hepatitis E, also called enteric hepatitis (enteric means related to the intestines) infection [1]. HEV is an Orthohepevirus [3], with a single-strand, positive-sense RNA genome of around 7.2 Kb in length and flanked with short 5' and 3' non-coding regions (NCR) [4]. The HEV genome comprises three partially overlapped open reading frames (ORFs): ORF1, ORF2 and ORF3. The ORF1, ORF2 and ORF3 encode the non-structural polyprotein (pORF1), capsid protein (pORF2) and the pleotropic protein (pORF3) respectively [5]. Further studies led to the identification of a novel viral protein synthesized from an ORF within ORF1, which was named as ORF4. This newly identified ORF4 was first reported by Nair et al. [6] which is exclusive to HEV G1. The indispensability of ORF4 in viral replication has been demonstrated. It has been revealed that ORF4 interacts with multiple viral and host proteins to enhance virus replication [6,7]. The expression of this ORF4 protein is regulated via an internal ribosome entry site (IRES)-like RNA element that is unregulated via cellular endoplasmic reticulum (ER) stress. ORF4 protein is rapidly turned over within cells as it possesses a proteasomal degradation signal [6]. Additionally, ORF4 has also been recognized in rats and ferrets [8,9]. Though ORF4 essentiality in G1 viral replication has been determined, its genetic and molecular characteristics remain to be explored.

Thus, in the present study we have analyzed the functional and structural characteristics of the ORF4 proteins by exploiting sequence-based bioinformatics methods. The data analysis of pathogen's genomic sequences has been progressively increased in the past few decades. At present, it is considered as an important approach in the epidemiology of infectious [10]. Availability of large number of complete genomic sequences of HEV on the (NCBI) has been achieved due to incredible effort made by researchers worldwide accumulating HEV data. This available data enabled us to comprehend the molecular basis of the evolution/ genomic variability/molecular biology in ORF4 region of HEV. In this context, a comparative codon-based characterization of the HEV ORF4 was conducted in an attempt to estimate the evolutionary divergence in the ORF4 gene sequences of HEV genome. The findings may contribute towards predicting the signature sequences based on the codon-based model of molecular evolution. Till date, specific treatment against HEV strains has not been discovered. Only Hecolin, a prophylactic vaccine is licensed only in China [11]. Thus, further studies are required for the development of specific drug molecule to treat HEV infections against all strains. Drug-discovery has been associated with intrinsically disordered proteins (IDPs) due to their prime features [12]. IDPs lack well-defined stable structure but are significantly involved in several biological processes, such as various signaling and regulatory processes, in protein-protein interactions (PPIs) [13-15]. Usually, IDPs form hub proteins in PPI networks [12]. Studies have reported the close association of various IDPs with several human diseases (tumor, Parkinson disease, Alzheimer disease, diabetes) [16-20]. The disease associated IDPs perform crucial roles in the disease through PPI networks [15]. IDPs undergo couple binding and folding end exist as ensembles of interconverting structures [21]. Due to the involvement in numerous PPIs, IDPs are considered as potential drug targets for drug molecules, which are capable of modulating or inhibiting their interactions, thus have opened tremendous potential in the field of drug discovery [22]. Very recently, the indispensability of ORF4 in HEV replication has been demonstrated [8]. In this context, we conducted computational analyses to provide an insight into the structural characteristics of this potential region. Therefore, the intrinsically disordered regions of the ORF4 proteins of HEV were analyzed. The findings obtained from the present analysis are augmented to envisage our understanding towards the biology of ORF4 protein of HEV.

## Materials & Methods:

### Sequence data acquisition:

The HEV ORF4 sequences were obtained from the National Centre for Biotechnology Information (NCBI) GenBank (https://www.ncbi.nlm.nih.gov/genbank/) public database. The complete detail of the sequences considered for the present analysis is listed in Table 1 (see PDF).

### Multi-sequence alignment of ORF4 protein genes:

The ORF4 sequences considered for the present analyses were categorized into three datasets. Dataset I consisted of study sequences from the host organism Human. Dataset II contained study sequences from the host organism Rat. Dataset III contained study sequences from the host Ferret. The alignment for all three datasets was achieved using Clustal X2 in BioEdit v.7.2 [23].

### Mutational analysis of ORF4 protein genes:

Bioedit software was used to predict the amino acid substitution in the HEV study sequences encompassing human, rat and ferret.

### Analysis of entropy of ORF4 protein genes:

The Shannon entropy analysis of ORF4 was carried out using BioEdit software [23], for the identification of possible variability/mutability. The entropy of aligned amino acids sequences was calculated at particular odon position to comprehend the variation within these genes.

### Selection pressure analysis of ORF4 protein genes:

Mutation rates were determined for ORF4 gene sequences using Gene selection pressure. Gene selective pressure was estimated by Tajima test of neutrality implemented in MEGAX software [24]. Positive selection was considered when D value is found to be positive (greater than 0). The test compares the average number of nucleotide differences between pairs of sampled sequences (referred to as pairwise difference-) and the total number of polymorphic sites (segregating site- S) in the sampled DNA sequences. The difference in the expectations for these two variables (which can be positive or negative) defines the Tajima's D test statistic. A positive Tajima's D signifies selection while negative D specifies purifying selection.

### Codon degeneracy patterns estimation of ORF4 protein genes:

The estimation of different codon values including nucleotide diversity (π), number of segregating sites (S) and transition to transversion ratio (R) in ORF4 gene sequences was undertaken for all the datasets. The analysis was conducted using the MEGAX software [24].

### Structural analysis of ORF4 proteins

Recent study on intrinsic disordered proteins (IDPs) revealed that they have the potential to act as drug targets [17-20]. Therefore, we evaluated the different structure levels of ORF4 proteins, obtained from different sources, to shed some light on its sequence composition, secondary structure elements, intrinsic disorder content and binding tendency. Thus, a set of different computational prediction methods was exploited to determine the stricture of ORF4 protein.

PSIPRED (http://bioinf.cs.ucl.ac.uk/psipred/), SOPMA (Self-Optimized Prediction Method with Alignment) (https://npsa-prabi.ibcp.fr/cgi-bin/npsa_automat.pl?page=/NPSA/npsa_sopma.html) and PONDR-fit (http://original.disprot.org/pondr-fit.php), TASSER (Iterative Threading ASSEmbly Refinement) [25] and PROCHECK [26]. The structural analyses were conducted using four different ORF4 protein sequences, i.e., LC057248 (HEV) KU168733 (Human), JN167538 (Rat) and LC177791 (Ferret).

## Results and Discussion:

To study the structure and function of protein, in-silico analyses have become a very valuable method [27]. Recently, analysis on proteins using in-silico tools has provided a huge contribution to the field of computational biology in elucidating the protein's functional and structural aspects [28,29]. In this context, we exploited different computational tools to reveal significant information on the ORF4 proteins of HEV.

## Analysis of mutations in ORF4 protein genes:

RNA viruses mutate at a very high rate, i.e., 10-6 to 10-4 new base substitutions per nucleotide per cell. Additionally, it has been well documented that virus with single-stranded genome appears to mutate faster than double-stranded viruses [30]. For mutational analysis, the sequence NC_038504/ Germany/2009, KU168733/India/2013 and JN998607/Netherland/2010 was used as a reference genome for dataset I, II and III respectively. The predicted mutations in the ORF4 genomes for datasets, i.e., Human, Rat and Ferret are summarized in Table 2 (see PDF). Our mutational analysis mostly showed changes in ORF4 genes, which corresponded to both synonymous and non-synonymous mutations (Figure 1 - see PDF). Thus, it can be interpreted that HEV also exhibits a high degree of genetic variation like other RNA viruses, due to viral RNA polymerase non-proofreading activity, rapid rates of replication, immense population size, and immunological pressure [30]. Thus, the previous hypothesis suggesting high mutation rates in RNA viruses substantiate our findings.

## Analysis of entropy in ORF4 protein sequences:

The entropy is one useful method of quantification of diversity in amino acid sequences [31]. Structurally or functionally important amino acid variations are correlated with high scoring entropy values [32]. The entropy analysis revealed a total of 1, 63 and 23 sites were identified in datasets I, II and III for Human, Rat and Ferret respectively (S1 Table - see PDF). The entropy percentages for ORF4 genomes are as follow: Human: 0.006% (1/159), Rat: 0.342% (63/184) and Ferret: 0.125% (23/184) respectively. Therefore, ORF4 genomes in rat observed the largest variation followed by ferret genomes and human genomes had the least variation (Figure 2 - see PDF). However, further thorough experimental investigations in conjunction with other studies (site directed mutagenesis) are mandatory to establish relationships between the reported mutations and their corresponding functional changes. Moreover, detailed insights into the mechanism of these strains are needed to confirm their pathogenicity and zoonotic potential.

## Analysis of positive selection in ORF4 protein genes:

Gene selective pressure for genes was estimated using the Tajima's Neutrality Test. The results suggested that genes comprising dataset I was found to be under purifying selection as indicated by negative D value, i.e., -1.093. However, dataset II and III consisted of genes under positive selection as indicated by positive D values, i.e., 0.861 and 0.554 for Rat and Ferret respectively. The selection pressure revealed the prevalence of positively selected sites in datasets II and III. While prevalence of purifying selection in Human dataset was observed. This suggests that the ORF4 region evolution is mainly driven by positive selection in Rat and Ferret. Thus, prevalence of non-synonymous mutations with high entropy scores corresponding to positively selected sites in Rat and Ferret datasets suggested high variability in these ORF4 protein genes.

## Estimation of codon degeneracy patterns between hosts in ORF4 protein genes:

The variation in codon properties was examined due to the codon degeneracy that was maintained in ORF4 protein genes. Nucleotide diversity (Π): The least nucleotide diversity in codon pattern was observed in Humans, and maximum divergence was found in Rat. The value of Ferret was intermediate between Human and Rat. The codon patterns followed the order of nucleotide divergence in the order Rat > Ferret > Human (Table 3 - see PDF).

## Number of segregating sites (S):

The estimated segregated site in the ORF4 was in accordance with the nucleotide diversity (Π). The highest S was correlated with highest Π value. The codon patterns followed the order of segregation sites in the order Rat > Ferret > Human (Table 3 - see PDF).

## Transitions more common than transversions (R):

The estimated transition/transversion bias for the hosts ranges from 0.3 to 5.5 in the ORF4 region. Rate of occurrence of transitional substitutions were much greater than the rate of transversion substitutions in all the natural hosts (Table 3 - see PDF). Higher transition/transversion ratio values in ORF4 region also reveals that less diversity in the amino acid composition due to less transversions, as more transversions which result in substantial dissimilar chemical composition [33]. Our results are in accordance with the previous study on HEV that suggested high transition to transversion ratio [34]. The phenomenon is mainly attributed to two mutually non-exclusive hypotheses: the mutational hypothesis and the selective hypothesis. The mutational hypothesis posits that transition rates are higher than the transversion mutation rates in both the coding and non-coding sequences [35,36]. The selective hypothesis holds that natural selection disfavors transversions [30,37]. Thus, our investigation showing biasness towards transition suggests that transitional mutations are more favored than transversions in the ORF4 region, which supports earlier mentioned hypotheses [34]. Thus, it can be interpreted that both mutation and natural selection influenced the ORF4 genomes. This is consistent with earlier report that revealed the co-existence of mutation-selection balance in RNA viruses [33].

## Analysis of structure of ORF4 proteins:

Earlier studies have revealed that a certain type of protein has been recognized with a lack of a well defined structure under physiological conditions but perform crucial biological functions. This class of proteins or protein regions are defined as intrinsically disordered proteins (IDPs) or intrinsically disordered protein regions (IDRs) [13,14]. An IDP possesses a unique feature, which enables it to interact with one to many and many to one signaling [38]. The significance of IDPs in biological functions, such as recognition, regulation, signaling, and protein-protein interaction (PPI) network control has been well documented [15]. IDPs are closely linked with human diseases (tumor, cardiovascular disease, neurodegenerative diseases, and diabetes) [16-20]. Due to IDPs involvement in diverse signaling and regulatory processes, strategies in drug discovery aiming at IDPs have gained momentum [15,22]. Therefore, these IDPs due to their unique structures act as potential targets in drug designing. Furthermore, IDPs are usually hub proteins in PPI networks, and PPIs are potential sources for drug targets. Thus, in this study we have examined the sequence and structure of ORF4 protein in order to reveal their prime features as a potential for drug target molecule. IDPs can be easily predicted by bioinformatical methods due to their peculiar amino acid composition [39-43]. Dunker and colleagues () [9] categorized amino acids into three groups based on their composition enrichment in ordered and disordered segments, i.e., the order-promoting group (C, W, Y, I, F, V and L), the disorder-promoting group (M, K, R, S, Q, P and E) and the neutral group (A, G, H, T, N and D) [44]. Initially, we performed a sequence-based comprehensive analysis of ORF4 proteins (LC057248, KU168733, JN167538 and LC177791) in terms of amino acid composition to elucidate their functional properties (Figure 3 - see PDF). Our results clearly revealed that all the ORF4 sequences were enriched in characteristic disorder-promoting residues (Arg, Pro and Ser) and neutral residues (Ala, Gly and Thr). Additionally, abundance of high proportion of structure-breaking residues (Gly and Pro) has been suggested that the protein is an IDP [45]. Also, the largest fractional change between the ordered and disordered protein is exhibited by Pro [46]. Thus, abundance of Pro amino acid residue in the ORF4 protein (KU168733), clearly indicated that the ORF4 protein (KU168733) particularly contains significant fraction of intrinsic disorder in comparison to other ORF4 proteins LC057248, JN167538 and LC177791. After the initial primary structure analysis, the secondary structure elements were determined that showed the presence of all three major contents including alpha helix, beta-strand and coils (S2 Table - see PDF). However, it was evident from our results that the ORF4 protein obtained from human (KU168733) was characterized with prevalence of coils and lack of secondary structure elements (helix and sheet) in comparison to other ORF4 proteins (S2 Table - see PDF). Protein-protein interactions (PPIs) are considered as potential sources for drug targets [22]. Intrinsic disorder is utilized in protein-protein interactions: namely, one disordered region binding to many partners and many disordered regions binding to one partner [38]. Therefore, we analyzed the predisposition of intrinsic disorder of ORF4 proteins. Based on predicted percentage of intrinsic disorder (PPID) in ORF4, the ORF4 sequences were classified into different protein variants: Ordered proteins (ORDPs); Intrinsically disordered protein regions (IDPRs); and Intrinsic disordered proteins (IDPs) [47]. The first category ORDP includes protein sequences, which have PPID less than 10%. The IDPR category includes protein sequences having PPID 10 - 30%. Lastly, the IDP category includes protein sequences, which are predicted to have PPID more than 30%. Thus, based on PPID, the ORF4 protein sequences considered in the study were categorized into different variants. The ORF4 proteins LC057248 (HEV) and LC177791 (ferret) were categorized into the intrinsically disordered protein regions (IDPRs), as they consisted of PPID in the range between 10% to 30% (Figure 4A and 4D - see PDF). The ORF4 protein JN167538 (rat) was categorized into ORPDs, as it consisted of less than 10% of PPID (Figure 4C - see PDF). The ORF4 protein KU168733 (human) was categorized into the IDPs as it consisted more than 30% of PPID. It was observed that the major portion of the polypeptide chain of the ORF4 protein KU168733 was highly disordered, revealing it as an IDP (Figure 4B - see PDF). Thus, taken altogether, beginning from the initial sequence analysis, secondary structure element up to fraction of intrinsic disorder content, it is clearly revealed that the ORF4 protein obtained from host human possesses the attributes of an IDP. IDPs perform significant roles in recognition, regulation, signaling, and protein-protein interaction (PPI) network control, thus are considered as potential targets in structure-based drug designing. Moreover, IDPs generally represent themselves as hub proteins in PP1 networks, and PPIs are potential sources for drug targets [15,22].

Furthermore, the generated 3D ORF4 protein models were comparatively visualized (Figure 5 - see PDF). Compared with other ORF4 proteins, i.e., JN167538 (ORDP), LC057248 and LC177791 (IDPRs), the ORF4 protein KU168733 (IDP) 3D model possessed a highly flexible and random coiled-like structure (Figure 5B - see PDF), which shows consistency with the previous report suggesting IDPs fail to arrange into a definite 3D structure under physiological conditions due to increased level of disordered-promoting residues [44]. Thus, out of several models, the obtained model from host Human can be considered as a reliable drug target due to its characteristic highly disordered (IDP) structure [15-20, 22]. Additionally, identification of clefts, tunnels and pores accessible to ligand molecules is essential in the context of structure-based drug design process [48,49]. Thus, the modelled structure of ORF4 protein (KU168733) was scrutinized using PDBsum analysis to reveal the presence of binding sites. Interestingly, the modelled ORF4 protein revealed the presence of 10 clefts (S1 Figure - see PDF), which determines their interaction with other molecules [50]. Clefts or pockets present on protein's surface are sizeable depressions that have tendency to be enzyme active sites [48]. Thus, to sum up our observations it can be interpreted that ORF4 protein (KU168733), due to its characteristics of an IDP, i.e., prevalence of Gly, Pro and Ser, lack of secondary structure with the predominance of coils, in addition to presence of several clefts, suggest its commitment towards interaction with other target molecules. Thus, it can be considered as a reliable drug target.

## Conclusion:

This novel study was aimed to collect information and discusses in the ORF4 of HEV. It provided detailed analysis on the occurrence of genomic diversity in the ORF4 protein genes of HEV. Further, the ORF4 protein of HEV was analyzed at different structural levels to shed light on its putative functions. Our presented results on function and structure of HEV ORF4 are theoretical hypotheses. Therefore, validations involving ORF4 structure by both computational and experimental approaches are further required.

## Funding:

Not applicable

## Author's contributions:

SP conceptualized the research. SP and ZS designed the manuscript. ZS was a major contributor in writing the manuscript and performed the biocomputational analysis of the protein. KP and AA proofread the manuscript. All the authors read and approved the final manuscript.
